# Driving forces of land surface temperature anomalous changes in North America in 2002–2018

**DOI:** 10.1038/s41598-020-63701-5

**Published:** 2020-04-24

**Authors:** Yibo Yan, Kebiao Mao, Jiancheng Shi, Shilong Piao, Xinyi Shen, Jeff Dozier, Yungang Liu, Hong-li Ren, Qing Bao

**Affiliations:** 1 Institute of Agricultural Resources and Regional Planning, Chinese Academy of Agricultural Sciences, Beijing, 100081 China; 2 School of Geography, South China Normal University, Guangzhou, 510631 China; 3 State Key Laboratory of Remote Sensing Science, Institute of remote sensing and Digital Earth Research, Chinese Academy of Science and Beijing Normal University, Beijing, 100086 China; 4 College of Urban and Environment Sciences, Peking University, Beijing, 100871 China; 5 Civil and Environmental Engineering, University of Connecticut, Storrs, CT 06269 USA; 6 Bren School of Environmental Science & Management, University of California, Santa Barbara, CA 93106-5131 USA; 7 Laboratory for Climate Studies & CMA-NJU Joint Laboratory for Climate Prediction Studies, National Climate Center, China Meteorological Administration, Beijing, 100081 China; 8 Institute of Atmospheric Physics, Chinese Academy of Sciences, Beijing, 100029 China

**Keywords:** Attribution, Climate-change impacts

## Abstract

The land surface temperature (LST) changes in North America are very abnormal recently, but few studies have systematically researched these anomalies from several aspects, especially the influencing forces. After reconstructing higher quality MODIS monthly LST data (0.05° * 0.05°) in 2002–2018, we analyzed the LST changes especially anomalous changes and their driving forces in North America. Here we show that North America warmed at the rate of 0.02 °C/y. The LST changes in three regions, including frigid region in the northwestern (0.12 °C/y), the west coast from 20°N–40°N (0.07 °C/y), and the tropics south of 20°N (0.04 °C/y), were extremely abnormal. The El Nino and La Nina were the main drivers for the periodical highest and lowest LST, respectively. The North Atlantic Oscillation was closed related to the opposite change of LST in the northeastern North America and the southeastern United States, and the warming trend of the Florida peninsula in winter was closely related to enhancement of the North Atlantic Oscillation index. The Pacific Decadal Oscillation index showed a positive correlation with the LST in most Alaska. Vegetation and atmospheric water vapor also had a profound influence on the LST changes, but it had obvious difference in latitude.

## Introduction

land surface temperature (LST) is an important reference index used to measure the land environment and has important impacts on regional material and energy cycles, ecological system balance and human production and life^1–3^. Regional LST will incur sensitive and obvious changes with the differences in time and space^4^. In particular, annual change in LST have large impacts on human development. For example, in July 2018, Canada experienced a series of high temperatures not seen in decades that killed at least 70 people in Quebec. In July 2017, the west coast of the United States was hit by a heat wave that broke 100-year records in many places and caused frequent wildfires in many western states. These are serious threats to human life and economic development. North America has rich climate types and complex geographical environment, and its LST changes have great research value. The Cordillera mountain system in the west has a lot of glacial snow, and the glacier melt water in this region is closely related to the LST^5^. Moreover, this region is located at the junction of the Pacific plate and the American plate, with frequent geological and volcanic activity^6^, and the variation in LST can reflect the energy changes from underground activities to some extent^7^. The central region of North America is an important agricultural area that is extremely vulnerable to the interactions between hot and cold air currents from the Arctic Ocean and the Gulf of Mexico due to the very large corridor formed by the mountains to the east and west. The plateau/mountainous region in eastern North America is affected by the uplift of moist airflow in winter and summer^8^, and meteorological disasters such as blizzards and rainfall are frequent. Most areas of Greenland in the northeast of North America are located in the frigid zone, and more than 80% of the whole island is covered by snow and ice, which plays an important role in stabilizing the global climate environment^9^. In summer, southern North America is extremely vulnerable to tropical cyclone activities^10^, which results in hurricanes, rainstorms, floods and other major disasters and causes great harm to local economic production and development. In recent years, a new hot issue has emerged regarding the annual change in global and region temperature, which is the global warming^11–14^ that has been of concern for a long time. Climate warming will lead to global precipitation redistribution, melting of glaciers and permafrost, sea level rise, frequent occurrence of extreme disasters and other problems, not only harming the balance of natural ecosystems but also threatening the survival and development of mankind^14–16^. This has become a major issue for the development of many countries and regions. These phenomena, including climate warming, glacial meltwater, meteorological disasters, volcanic earthquakes, and land cover change, are closely related to LST changes. Paying close attention to the abnormal LST changes and exploring the reasons for the changes have great importance on strengthening disaster monitoring and early warning, protecting agricultural production, promoting ecological protection and maintaining human daily life.

Higher-quality data can help monitor and analyze temporal and spatial variations in LST more accurately. Traditionally, people mainly measure LST by ground stations. This kind of data can accurately reflect the LST near the stations with high precision and strong reliability^17^. Moreover, data from ground stations are less disturbed by cloud and rain weather, so the data integrity is relatively high and the time series covered is long^18^. Many researchers have made long time series of observations of regional and global LST using statistical data from ground stations^19,20^. However, the distribution of stations is usually sparse, and some areas with rugged terrain and harsh environments even have no stations, resulting in a lack of spatial continuity of the data, so it is difficult to reflect the spatial difference of the data. With the progress of information technology, many authoritative institutions in the world use station data from different systems to carry out data assimilation interpolation and generate reanalysis data with greater spatial continuity^21–23^. Although this kind of data improves the deficiencies of station data to some extent, the spatial resolution of reanalysis data is usually low. And the data accuracy in some areas needs to be improved, so it is still difficult to meet the requirements for more accurate spatial analysis. Since the 1970s, with the development of satellite launch technology and sensor technology, remote sensing has become a new means to monitor LST. The characteristics of a large observation range, good timeliness and good spatial continuity of remote sensing LST data can improve the limitations of traditional station data and better display spatial changes in surface information, and this data is suitable for spatial analysis on a large scale^24–27^. However, compared with the ground station data, remote sensing data also has its own limitations. The most important manifestation is that remote sensing signals are impacted by cloud, rain and other weather interference when propagating through the atmosphere, so there are always some pixels of missing information and low precision in remote sensing images. Among remote sensing sensors, MODIS (Moderate Resolution Imaging Spectroradiometer) is mounted on the Terra and Aqua satellites, which were launched in 1999 and 2002, respectively, and the LST data from MODIS are widely used because of the good temporal and spatial resolution^28^. Although the MODIS LST data is relatively mature, some areas image still suffer from a lack of information and low accuracy due to the interference of atmospheric conditions, which greatly reduces the quality and utilization rate of remote sensing data and causes great difficulties for subsequent analysis of relevant issues^29^. To solve this problem, many researchers have tried to improve the accuracy and integrity of the data by improving inversion models^30^, cloud detection technology^31^, data repair^32^ and so on.

In recent years, some natural disasters in North America such as fire, drought, earthquake and other have occurred frequently, and LST is one of important physical parameters for the characterization of these disasters. This study mainly used remote sensing data to reveal the variation of LST from the pixel scale, especially some anomalous changes in North America. Firstly, ground stations data and adjacent pixels were used to reconstruct remote sensing LST data with more complete information by the interpolation model. Secondly, we analyzed the LST changes in different North American regions from 2002 to 2018 on pixel scale, especially focusing on anomalous areas with obvious linear variation trends. Finally, we combined land surface, atmosphere, ocean and other parameters and used multiple methods to analyze the driving factors that lead to spatiotemporal variations in LST. Through analysis, we obtained the anoumalous variation areas and some driving factors of LST in North America in recent years, which could help us understand factors of regional climate change, and provide meaningful conclusions and basis for agricultural production, disaster monitoring and early warning, and ecological protection.

## Results

### More accurate and complete LST data of North America from 2002 to 2018

**Figure 1:**
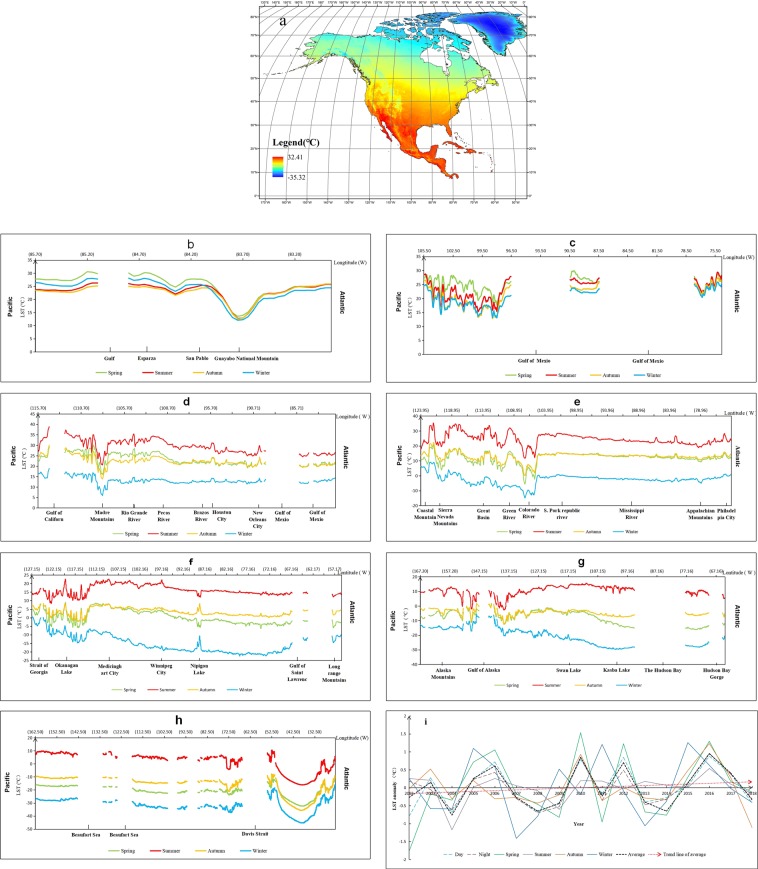
Average LST and its variations in North America from 2002 to 2018. (**a**) Average LST for all years in North America from 2002 to 2018. (**b–h**) The average LST in different seasons at 10°N, 20°N, 30°N, 40°N, 50°N, 60°N and 70°N. (**i**) Variation in LST anomalies in different time dimensions in North America from 2002 to 2018.

The distribution of LST in North America has strong spatial differences. The average LST in North America was 1.92 °C, and the maximum spatial difference in LST was 67.73 °C (Fig. 1a). The California Peninsula on the west coast was the region with the highest annual average LST of 32.41 °C, and it is a world-famous dry heat region. The lowest annual average LST was found in the interior of Greenland, reaching −35.32 °C, which was mainly caused by the location at higher latitudes and deep inland. To more clearly and concretely reveal the spatial differences in LST in North America, we performed a specific analysis of the LST in different seasons in the area traversed by the seven latitude lines of 10°N (Fig. 1b), 20°N (Fig. 1c), 30°N (Fig. 1d), 40°N (Fig. 1e), 50°N (Fig. 1f), 60°N (Fig. 1g) and 70°N (Fig. 1h). We found that under the influence of topographic conditions, the spatial difference in LST in the western part of North America was large, and that in the central and eastern parts of North America was relatively small. The valley areas in the western Alpine region had a higher LST than the surrounding areas and were major areas of agricultural production and population urban development, such as the Central Valley between the Sierra Nevada and the coastal mountains (Fig. 1c). In the middle and high latitudes, the LST showed obvious temperate climate characteristics, especially in the inland areas of the middle and high latitudes. Under the influence of the differences in the hydrothermal properties of land and water, the LST difference between Nippon Lake and the surrounding area showed opposite variation characteristics in the cold season and warm season (Fig. 1d). Influenced by this phenomenon, the temperate inland lakes and rivers are the seasonal migration areas for many animals, such as Swan Lake at the latitude of 60°N (Fig. 1d). In the western mountain region of the middle and high latitudes, the spatial difference in LST was extremely significant. In this area, glaciers, river valleys, volcanoes and other staggered distributions and landscape types are extremely rich, which makes this region a world-famous tourist resort area. These spatial characteristics of LST in different areas are of great significance for agricultural production, ecological environmental protection and human development. After the analysis of spatial variation, we briefly analyzed the trend of LST from different time dimensions by anomalies of LST (Fig. 1i). The LST anomalies by day, night, different seasons and whole year showed similar trends of fluctuating increases. From the average LST of the whole year, the interval between two adjacent minimum of LST anomaly was a period of fluctuation, and each period lasted approximately 4–5 years. In 2016, the average LST reached its peak state in recent years, and the LST declined year by year since then.

### Variation trends of LST in different regions

**Figure 2:**
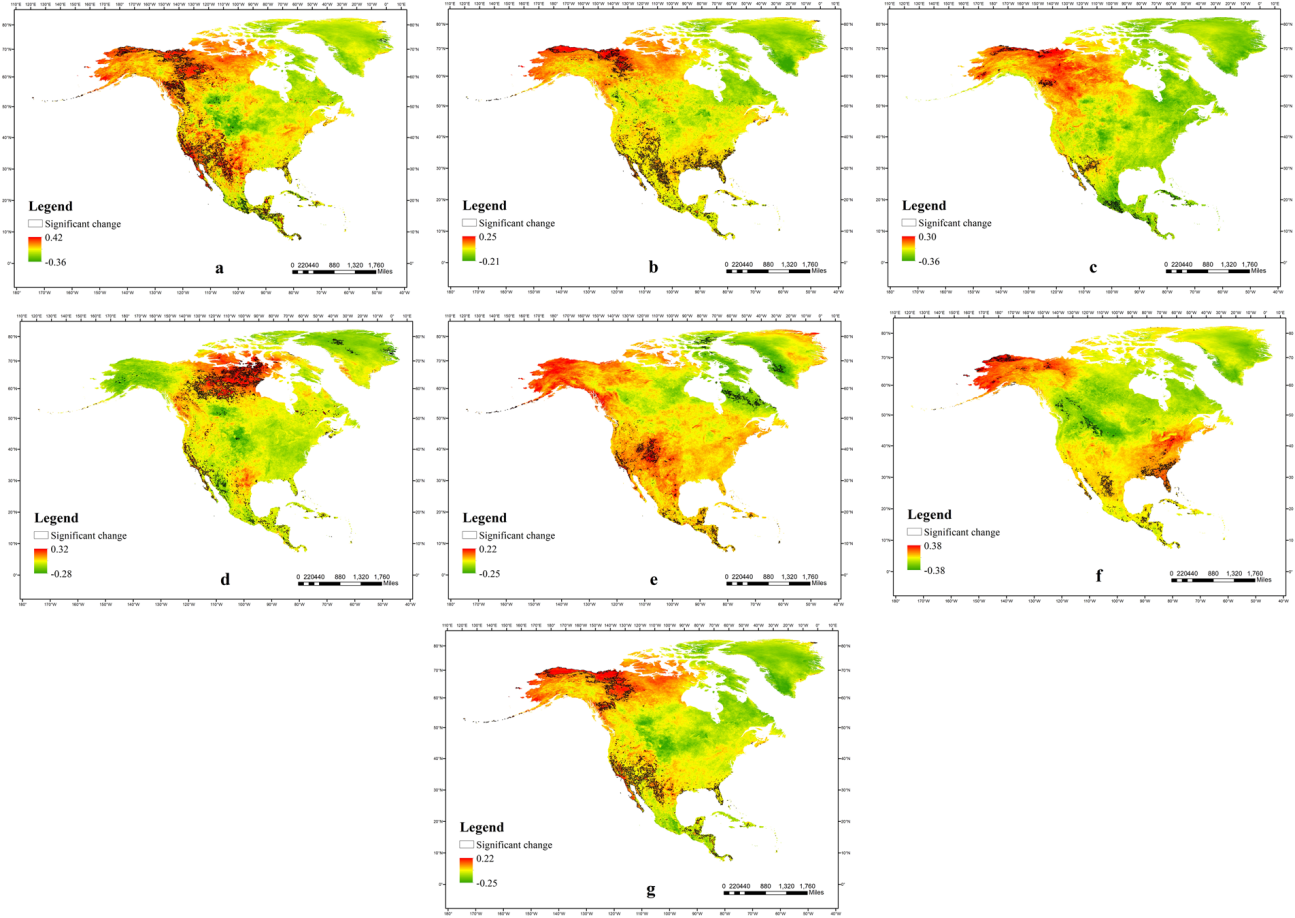
The interannual changes in LST in different time dimensions from 2002 to 2018. (**a,b**) The rate of LST change during day and night in North America. (**c–f**) The rate of LST change during spring, summer, autumn, and winter in North America. (**g**) The rate of LST change during the whole year in North America. The areas with black boundaries in the figures indicate that these regions passed the two-tailed t test with a confidence of 0.05, which represent a strong linear warming or cooling trend.

We comprehensively analyzed the linear variation in LST through the interannual variation rate of LST (Fig. 2) (see Methods) and focused on the regions with an obvious linear variation trend (The two-tailed t test with a confidence level of 0.05 was performed). We found a number of noteworthy regions in North America with abnormal changes. The west coast from 20°N–40°N was the region with the most abnormal LST changes in North America, and there was a significant linear warming trend (0.07 °C/y)in average LST. In this region, the area of LST increases in the daytime was much wider than that in night (Fig. 2a,b). In particular, nearly 30% of the California peninsula had a linear increase in daytime LST, with a rate of 0.08 °C/y (Fig. 2a). From the perspective of seasons, we found that this region had different degrees of warming trends in different seasons, and the distribution of warming in the region showed obvious differences in different seasons. In the region, the warming area in the cold season (spring and winter) was mainly distributed in the south (Fig. 2c,e), while the warming area in the warm season (summer and autumn) was mainly distributed in the north (Fig. 2b,d). Moreover, the warming area was larger in the warm season than in the cold season, and the warming rate was also larger. In addition to this region, the frigid region in the northwestern North America also had a significant warming trend, which was mainly reflected in the cold belt of Alaska and Canada. The range of LST increases in this region was very large, with the rate of annual LST reaching 0.12 °C/y (Fig. 2g). In the tropical regions south of 20°N, the annual average LST growth trend in recent years was also significant, with the rate reaching 0.04 °C/y (Fig. 2g). These three regions were the regions showing significant LST changes in multiple time dimensions. The percentages of areas with significant linear warming, significant linear cooling and non-significant change in different seasons are shown in Table 1. It could be found that the significant warming area was the most widely distributed in summer and the significant cooling area was the most widely distributed in autumn. The regions of significant warming were wider than the regions of significant cooling over the seasons.

**Table 1:** The proportion of LST variation in North America in different seasons.

Variable	Spring	Summer	Autumn	Winter
Significant warming	1.03%	4.72%	0.88%	1.78%
Significant cooling	0.28%	0.32%	0.49%	0.23%
Non-significant change	98.69%	94.96%	98.63%	97.99%

Other areas of North America also experienced significant LST changes, but they had strong seasonal difference. In summer, there was a significant warming in the northern and central parts of Canada (0.17 °C/y) (Fig. 2d). There was a significant cooling trend (−0.16 °C/y) on both sides of Davis strait in the autumn (Fig. 2e). In winter, there was a significant warming trend in Florida (0.16 °C/y) and a cooling trend (−0.23 °C/y) in Montana and Wyoming (Fig. 2f). Along the tropical eastern Pacific coast south of 20°N, LST showed completely opposite trends in different seasons. In spring, the region showed a significant cooling trend (−0.07 °C/y) (Fig. 2c), while in autumn, the region showed a significant warming trend (0.06 °C/y) (Fig. 2e).

### The influence of land surface and atmospheric circulation on LST

**Figure 3:**
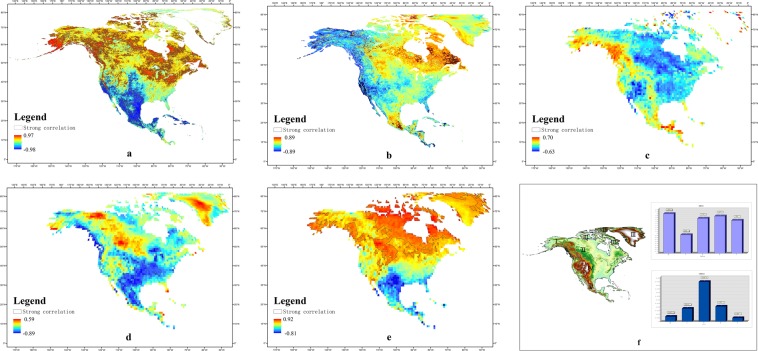
Correlation analysis of LST and other surface and atmospheric parameters. (**a**) Correlation coefficient between LST and NDVI. (**b**) Correlation coefficient between LST and SM. (**c**) Correlation coefficient between LST and AOD. (**d**) Correlation coefficient between LST and cloud fraction. (**e**) Correlation coefficient between LST and atmospheric WV content. The area highlighted with black boundaries in figures (**a**) to (**e** indicate that the absolute value of the correlation coefficient is greater than 0.6. (**f**) Simulation results of LST in different regions. The map of the partition was generated by Arc gis10.2 (https://www.arcgis.com)).

The reception of the land surface to solar radiation is influenced by atmospheric conditions and land cover. The correlations between NDVI (normalized differential vegetation index), SM (soil moisture), AOD (aerosol optical depth), cloud fraction and atmospheric WV (water vapor) and LST were analyzed (see Methods). When the absolute value of Pearson coefficient was greater than 0.6, we highlighted the pixels, indicating a strong correlation between a kind of parameter and LST. There was a strong correlation between LST and NDVI (Fig. 3a), but it had obvious zonal differences in latitude. LST was negatively correlated with NDVI in the area south of 40°N and positively correlated with NDVI in the area north of 40°N. The correlation between LST and SM was weaker than that with vegetation. The LST and SM were negatively correlated in the western California peninsula and positively correlated in the eastern Labrador peninsula (Fig. 3b). The correlation between LST and AOD was the weakest among all the factors in this study, and the correlation had no obvious distribution pattern (Fig. 3c). LST and cloud fraction were negatively correlated in most areas but positively correlated in a few areas. Moreover, the negative correlation was very significant, especially between 20°–40°N (Fig. 3d). The correlation between LST and atmospheric WV was very strong (Fig. 3e). This correlation had obvious zonal differences in latitude, showing a negative correlation in the region south of approximately 40°N, a positive correlation in the northern region. On the whole, among the five parameters selected in this study, the correlations between atmospheric WV and NDVI and LST were the strongest, followed by that of cloud fraction, and the correlations between SM and AOD and LST were the weakest.

On the basis of correlation analysis, we analyzed the regression relationships between LST and 5 kinds of parameters, latitude and altitude and studied the influence of these 7 factors on the spatial differences in LST in different regions of North America. We used these values to construct five regression models of LST with the 7 factors in 5 regions (Fig. 3f). On this basis, we randomly selected some points in the same image and simulated the LST values of these points by using the regression model built for each region (The distribution of sample points and verification points and the specific parameters of the model were given in the Supplementary Information). The LST regression models we established can simulate the regional LST well (R^2^ > 0.8) (All models and coefficients passed the two-tailed t test with a confidence of 0.05) (Fig. 3f). RMSE (Root Mean Square Error) was used to show the difference between simulated and predicted values, and NMRSE (Normalized RMSE) was used to show the variation of simulated differences within the region. For North America as a whole, latitude and altitude directly affect the amount of solar radiation energy received in the region and the inverse radiation intensity of the atmosphere, which are the most important factors affecting the spatial differences in LST. Atmospheric WV and NDVI also have great impacts on LST, while the other factors have relatively small impacts on LST. The RMSE between the LST values in different regions simulated by the 7 kinds of parameters and the original remote sensing LST values was within 2 °C. The simulation error for the polar islands was the largest, reaching 1.99 °C, while the simulation error for the Alaska region was less than 1 °C. The NRMSE was relatively small in the tropical and cold zone, while relatively large in the temperate zone, which was mainly related to the spatial difference of LST in different regions. By comprehensive correlation analysis and regression analysis, it could be determined that the vegetation index and atmospheric WV had a great influence on LST. Variations in regional surface vegetation and atmospheric WV cause changes in LST to some extent.

**Figure 4:**
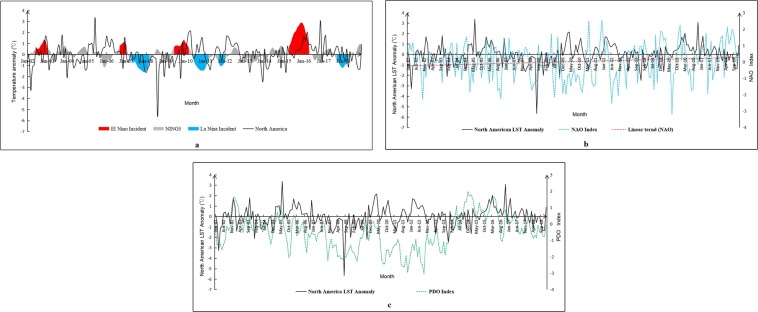
Overall variation trends of the LST anomaly and three climate indexes. (**a**) The variation in the monthly LST anomaly in North America and the SST anomaly in the NINO3 area from 2002 to 2018. (**b**) The variation in the monthly LST anomaly in North America and the NAO index. (**c**) The variation in the monthly LST anomaly in North America and the PDO index.

North America is surrounded by sea, and large marine climate activities have a great impact on it. We calculated the mean LST anomaly of North America from 2002 to 2018 and analyzed the consistency of LST anomaly with three indexs, including the SST (sea surface temperature) index of the NINO3 region (Fig. 4a), the NAO (North Atlantic Oscillation) index (Fig. 4b), and the PDO (Pacific Decadal Oscillation) index (Fig. 4c). In the past 17 years, there were four El Nino events during 2002/06–2003/01, 2006/09–2007/01, 2009/06–2010/03, and 2015/04–2016/04. Among them, the El Nino event that occurred between 2015 and 2016 was the most severe, lasting for a whole year, and the maximum NINO3 index reached 2.91 °C. There were four La Nina events in the past 17 years during 2007/04–2008/03, 2010/06–2011/03, 2011/09–2012/01, and 2017/09–2018/05. The LST in North America showed a periodic maximum with each El Nino event, while every La Nina event caused a periodic minimum LST in North America, indicating that El Nino and La Nina had a great influence on the variation in LST in North America. The LST in North America reached the highest level in recent years in 2016 under the influence of the strong El Nino event in 2015 and then fell sharply over the next two years. The consistency of the NAO index with the mean LST anomaly was relatively weak, and we found that the NAO had been enhanced in recent years (Fig. 4b). There was no obvious correspondence between the mean LST anomaly of North America and PDO index (Fig. 4c).

**Figure 5:**
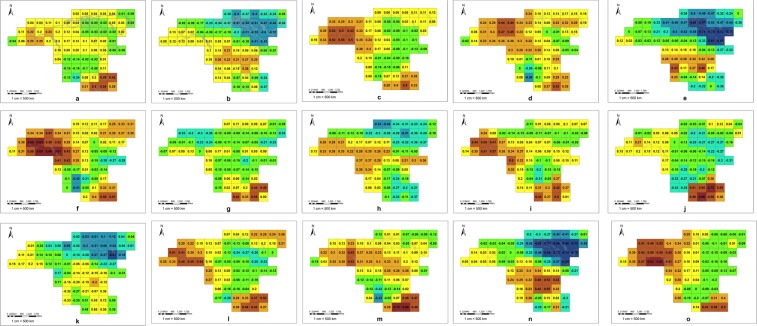
The correlation between the LST anomaly and three climate phenomena indexes. (**a–c**) The correlation between the LST anomaly and the index of NINO3, NAO, and PDO for the whole year. (**d–f**) The correlation between the LST anomaly and the index of NINO3, NAO, and PDO in spring. (**g–i**) The correlation between the LST anomaly and the index of NINO3, NAO, and PDO in summer. (**j–l**) The correlation between the LST anomaly and the index of NINO3, NAO, and PDO in autumn. (**m–o**) The correlation between the LST anomaly and the index of NINO3, NAO, and PDO in winter).

We analyzed the influence of three phenomena on local LST. The whole continent was divided into 71 grid units of 900 km*900 km, and the correlation coefficients of the NINO3 SST index, NAO index and PDO index with the mean LST anomaly in each region were calculated to analyze the influence of the three phenomena on the time variation of regional LST. We found that the SST index in the NINO3 region was positively correlated with LST in most areas of North America, and the positive correlation was very strong in southeastern Mexico and parts of Central America (r > 0.5) (Fig. 5a). The effects of the NAO on LST in North America were mainly reflected in the northeast, especially in Baffin Bay and Davis Strait, and there was a strong negative correlation between LST and the NAO index (r < −0.5) (Fig. 5b). The correlation between PDO and LST in North America was mainly reflected in the Alaska region in the northwest, and the PDO index showed a strong positive correlation with the LST anomaly (r > 0.5) (Fig. 5c).

Because the LST and circulation patterns all had significant seasonal differences, we analyzed the correlations between the three indexes and LST for different seasons (Fig. 5d–o). The areas influenced by the El Nino and La Nina phenomena were mainly distributed in the south of North America. The NAO had a great influence on the LST in northeastern North America. The influence of PDO was reflected in northwest North America. The areas influenced by the three phenomena in different seasons were relatively consistent with those of the whole year, but the intensity of the effect varied from season to season. These three phenomena had the strongest effect on LST in North America during winter and spring, followed by autumn and summer. In other parts of North America, these three phenomena also showed a strong correlation with regional LST. In winter and spring, there was a positive correlation between the NINO3 index and LST in northwestern North America (r > 0.4) and a negative correlation between LST and the NINO3 index in southwest of Mexico (r < −0.5). The correlation between LST in southeastern United States and the NAO index in winter and spring was also strong (r > 0.5). The PDO index showed a negative correlation with the southernmost parts of Mexico in spring (r < −0.5).

## Discussion

LST is a key variable for climate and ecological environment research. We reconstructed LST data to take advantages of the ground station data through building reconstruction model which overcome the effects of the cloud to some extent. This data has more complete coverage and higher data accuracy, which is very valuable for more accurate study of spatio-temporal variation of LST and monitoring of abnormal areas of LST. On this basis, we found some noteworthy areas of abnormal changes in LST, and explained some of the reasons for the changes.

**Figure 6:**
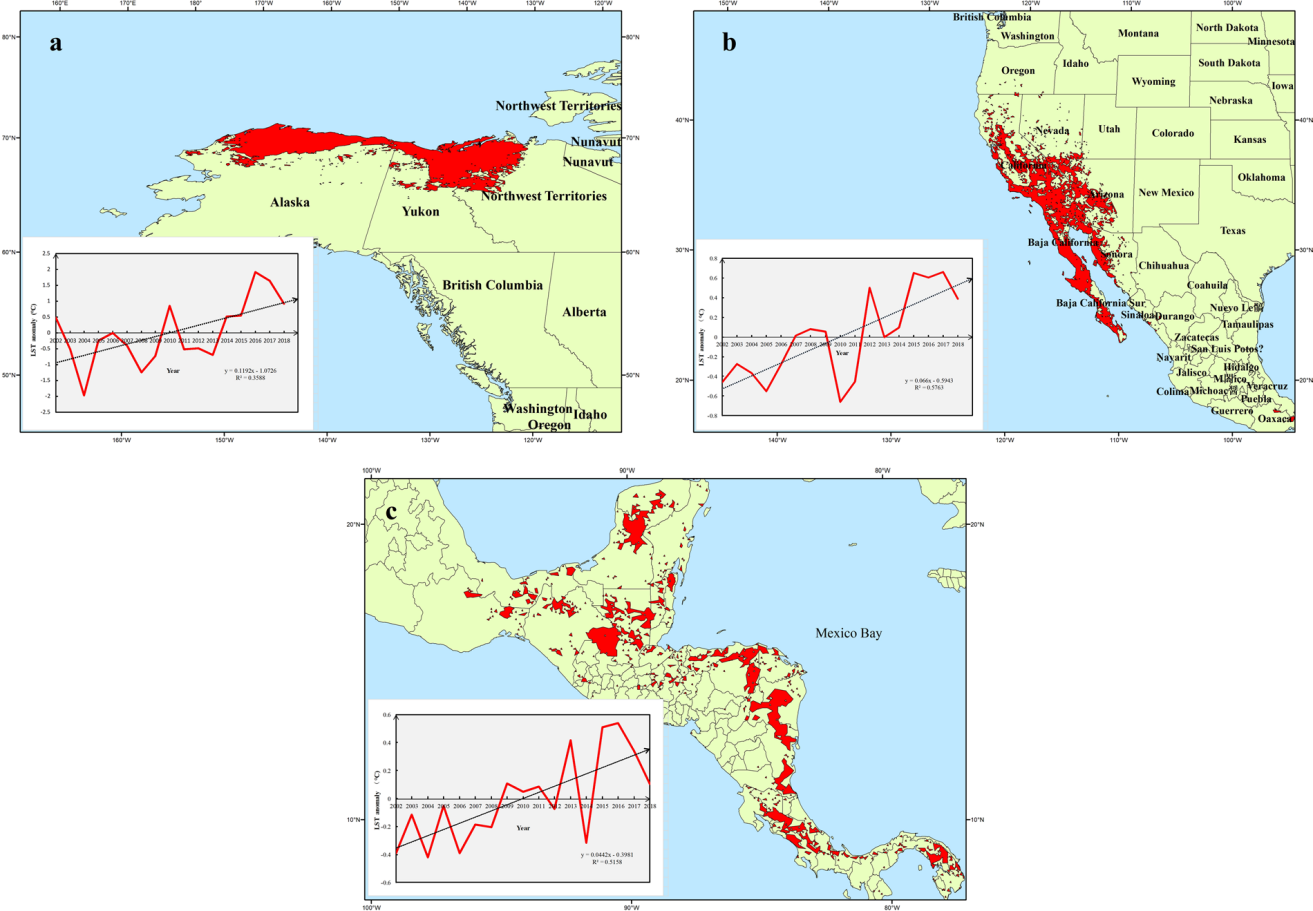
Mean annual LST anomalies of the three anomalous regions from 2002 to 2018. (**a**) The annual LST anomaly in the anomalous warming region of the frigid region in the northwestern from 2002 to 2018. (**b**) The annual LST anomaly in the anomalous warming region of the west coast from 20°N–40°N from 2002 to 2018. (**c**) The annual LST anomaly in the anomalous warming region of tropical areas south of 20°N from 2002 to 2018).

Overall, North America showed a volatility warming trend in recent years. The linear warming trends in the frigid zone in the northwest of North America was very obvious (Fig. 6a). Many scientists have performed a large amount of research on the climate and environment in this region and found some evidence of warming^33,34^. Our results suggested that warming trend was continuing in this region. As this region is located in the arctic cold zone, the resources of glacial snow and frozen soil in this region are relatively rich, which is very important for the balance and stability of the ecological environment in this region. A large continuous increase in LST is an important threat to the ecological environment in this region and must be considered. The LST in California peninsula in the western United States also showed a substantial and significant warming in recent years (Fig. 6b). And we found that in this region, the closer to the Pacific, the greater the rate of change in the LST was, which provied a new idea and basis for us to further explore the causes of the warming in this region. Coupled with the discovery of seasonal north-south movement of the warming regions, we believe that the abnormal trend may be caused by large scale natural factors. Due to the unique geographical features of this region, volcanic earthquakes and forest fires occur frequently in the region, so the abnormal warming trend in this region in recent years needs to be considered seriously. In addition, local residents should pay attention to the prevention of drought and fires, especially in the warm season (summer and autumn), because the area has a larger warming rate in these two seasons. What’s more, it is worth noting that this area belongs to the cordillera mountain system. Glacial meltwater is an important source of fresh water resources in this region, which is greatly affected by the sharp rise of regional LST. The warming of tropical areas south of 20°N is also noteworthy (Fig. 6c). The increase of LST in this area was also closely related to the drought and high temperature events.

The causes that affect LST change are complex and variable. Although we have not fully explained all causes of every abnormal regional changes, some important driving factors were found and we can make some new discoveries based on these conclusions. Through our research, we found that surface type and atmospheric conditions can affect changes in LST, especially vegetation and atmospheric moisture. We can even simulate the LST in some areas by some relevant factors of land surface and atmosphere, and this approach is helpful for data restoration and more comprehensive anomaly monitoring. However, compared with large climate activities, such as El Nino-La Nina, the NAO, and the PDO, the influence of land surface and atmospheric conditions on LST is relatively small. El Nino and La Nina are bound to cause periods of high and low temperatures in North America, respectively, and we found that this effect also has a certain lag period, about 1–3 months (Fig. 4a). It should be emphasized that not all periodic high and low temperatures are associated with El Nino and La Nina, for example, the LST anomalies in June 2005 and August 2009. The seesaw phenomenon of the NAO had a great influence on the LST changes in the eastern Atlantic coast of North America, especially in winter. The NAO index was negatively correlated with the LST in the northeast of North America and positively correlated with the LST in the Florida peninsula in the southeast of the United States. By observing the change trends of the NAO in recent years (Fig. 4b), we think that the cooling trend in northeastern North America and the warming trend in southeastern North America in winter are probably caused by the strengthening of the NAO. These results and discoveries have great significance to understand the forces of regional LST changes, the monitoring of abnormal regions, the early warning and prevention of disasters, and the protection and improvement of human life and production.

## Methods

### Research data

#### Data from remote sensing

MODIS is mounted on the Terra and Aqua satellites, which were launched in 1999 and 2002. The transit times of the two satellites were 10.30 am and 01:30 pm, respectively. MODIS has 36 discrete spectral bands, which can provide information reflecting land surface conditions, cloud characteristics, aerosols, surface temperature, ozone, ocean and other characteristics^35^. MODIS inversely generates LST by using information from middle infrared and thermal infrared bands. Many researchers have performed a large amount of research on the inversion algorithm and accuracy verification of LST^36–41^. The generalized split window method^39^ and day/night method^40^ are the official inversion algorithms of MODIS LST data at present. Through many verification analyses and improvements, the average precision of LST inversion for these two algorithms is approximately 1 K^42–44^. MODIS LST data have been widely used in many fields, such as climate change, water cycles, environmental assessment and agricultural production, due to the good temporal and spatial resolution and wide coverage^45,46^. The MOD11C3 LST products are inverted by the day/night method and obtained by projection, splicing, resampling and average synthesis.

The day/night method uses mid-infrared and thermal infrared band data during day and night to invert surface temperature and emissivity^40^. This method assumes that there is no significant difference in surface emissivity from day to night and that the angular form factor changes little in the region of interest in the middle infrared band. The model of surface temperature inversion by this algorithm is shown in Eq. (1)^40^. In this algorithm, N bands were measured twice (day and night), and the number of unknowns was N + 7 (emissivity of N channels, 2 surface temperatures, 2 moisture contents, and 1 mid-infrared channel); thus, the number of bands chosen must be greater than or equal to 7 to make the equation solvable. For MODIS, these seven bands are MODIS bands 20, 22, 23, 29, 31, 32, and 33.

1 $${\rm{L}}({\rm{j}})={\rm{t}}1({\rm{j}})\varepsilon ({\rm{j}}){\rm{Bj}}({\rm{Ts}})+{\rm{La}}({\rm{j}})+\frac{1-\varepsilon ({\rm{j}})}{\pi }.[{\rm{t}}2({\rm{j}})\alpha \mu 0{\rm{E}}0({\rm{j}})+{\rm{t}}3({\rm{j}}){\rm{Ed}}({\rm{j}})+{\rm{t}}4({\rm{j}}){\rm{Et}}({\rm{j}})]$$

In formula (1), L(j) is the radiation intensity of band j; ε(j) is the band emissivity; similar for B_j_(T_S_), L_a_(j), and E_0_(j); and E_d_(j) and E_t_(j) are the band-averaged solar diffuse irradiance and atmospheric downward thermal irradiance at the surface, respectively.

#### Data from ground stations

Although the MODIS LST data are relatively mature, some areas still suffer from a lack of information and low accuracy due to cloud interference, which affects the accuracy of spatiotemporal analysis. Therefore, considering the advantages and disadvantages of remote sensing data and ground station data, we repaired and reconstructed the MODIS monthly LST data with data from ground station LST and adjacent pixels to make the data more complete and accurate. Since 2000, most stations have been able to measure both air temperature and LST. Only a small part of the area can only measure air temperature due to mechanical equipment. For stations lacking of LST, we calibrated the air temperature of them using the difference between the MODIS LST and air temperature of the same station in the near data. Assuming that the difference does not change much between the LST and the air temperature at the same station in the near dates, then we use the air temperature to calculated LST by using the difference in the near date. The ground stations data we used are from the National Oceanic and Atmospheric Administration’s National Center for Environmental Information (NCEI) website. The NCEI’s land-based observations are obtained by instruments from all regions of the continent, including temperature, dew point, relative humidity, precipitation, wind speed and direction, visibility, atmospheric pressure, hail, fog, and thunder, and cover many kinds of time scales, including hours, days, months, and years. The distribution of ground stations in North America is shown in Supplementary Information. The ground stations are more densely distributed in the United States, central and southern Canada and some central American countries, while in the northern polar islands the distribution is sparse. In some areas and time, the ground station data has the phenomenon of missing value, mainly in the severe weather environment. We first selected the data from 4 time periods from 2002 to 2018 provided by the institution, including 01:00 am, 10:00 am, 1:00 pm and 10:00 pm, which approximately correspond to the four time periods of 01:30 am, 10:30 am, 1:30 pm and 10:30 pm in the MODIS LST data. We used the stations LST to interpolate the MODIS LST data at different times. After the restoration of the data, we reselected the monthly stations LST data from 2002 to 2018 provided by the NCEI to verify the accuracy of the reconstructed LST data.

#### Land surface and atmospheric parameters

The factors affecting LST are complex. Based on previous research results on the factors of LST^47–51^, we selected seven parameters, latitude, altitude, the NDVI (normalized differential vegetation index), SM (soil moisture), AOD (aerosol optical depth), cloud fraction and atmospheric WV (water vapor), to analyze the driving factors of spatial differences in LST. The status of vegetation on the surface directly affects the amount of solar radiation received by the surface, and the vegetation also affects the change of LST through climate regulation during the growth process. Soil moisture is an important reflection of water resources in the land surface and certain depth, and it can change the LST by adjusting the specific heat capacity of the land. Aerosol optical depth, Clouds and atmospheric water vapor, as important manifestations of atmospheric conditions, have important effects on the propagation and reception of solar radiation signals. The detailed data introduction are shown in Table 2.

**Table 2:** Introduction of others parameters about land surface and atmospheric.

Variable	Product	Spatial coverage	Temporal Resolution	Spatial Resolution	Source
NDVI	MOD13C2	Global	Month	0.05°	NASA
SM	AMSR-E, SOMS,AMSR-2	Global	Month	0.05°	JAXA, ESA
AOD	MOD08_M3	Global	Month	1°	NASA
Cloud fraction	MOD08_M3	Global	Month	1°	NASA
Atmosphere WV	MOD08_M3	Global	Month	1°	NASA

#### Parameters of climate phenomena

The ocean on the Earth’s surface accounts for approximately 71% of the global total area and is an important factor affecting the thermal distribution, atmospheric circulation, weather changes and climate differences on the land surface^52^. The El Nino and La Nina phenomena are large-scale phenomena of continuous temperature increase and decrease in the central and eastern equatorial Pacific Ocean. These phenomena affect the WV energy cycle between land and sea and have important impacts on the temperature and rainfall in many parts of the world^53^. They are the strongest signals of interannual changes in the climate system. In general, when the SST anomaly index in the area of NINO3 (5°N-5°S, 90° W-150°W) reaches more than 0.5 °C for 6 consecutive months, it is defined as an El Nino event. In contrast, if the SST anomaly index of this region is below −0.5 °C for 6 consecutive months, it is defined as a La Nina event. The NAO refers to the inverse relationship between the Azores high-pressure center and the Icelandic low-pressure center and is one of the most apparent North Atlantic atmospheric modes. It not only directly affects the climate of the North Atlantic and nearby areas but also has an important influence on the temperature and precipitation of the whole northern hemisphere. The NAO index can explain 31% of the variance in average winter temperature in the northern hemisphere over a period of time^54,55^. The Pacific Decadal Oscillation (PDO) is a Decadal cycle of Pacific climate change, and it is characterized by unusually warm or cold surface water temperatures in areas north of 20°N in the Pacific Ocean^56,57^. PDO has a huge impact on the climate of surrounding regions, including North America^58^. In this study, we used the NINO3 SST index, NAO index and PDO index to analyze the influence of these three phenomena on the time variations in LST in different areas of North America. All data of these three indexes are from the US National Climate Prediction Center (NCPC).

### Data repair methods

**Figure 7:**
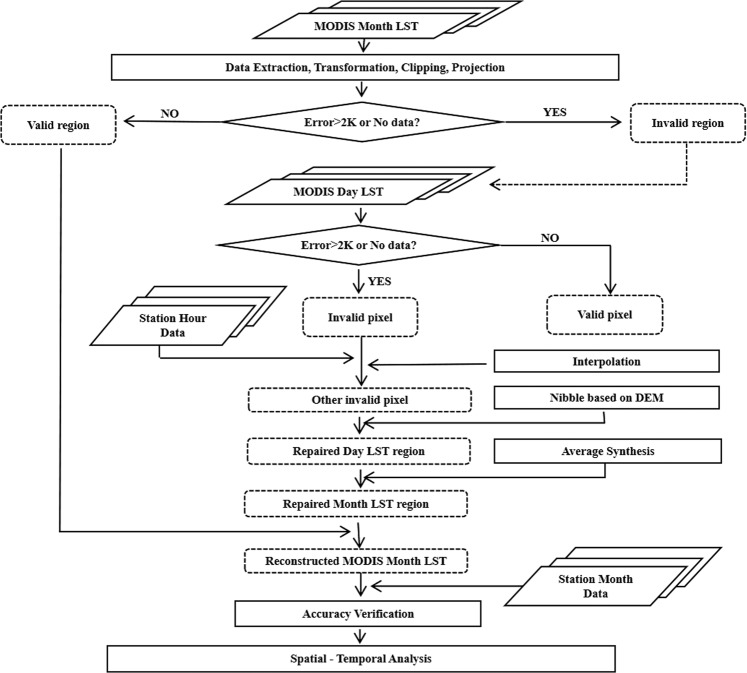
Technical flow chart of data repair.

The low accuracy of remote sensing data in some areas caused by cloud interference is a common problem faced by optical remote sensing^59^. This problem affects the accuracy of data analysis, and many researchers use different data repair methods to address this problem. These methods can be broadly divided into three categories depending on the data source used for the fix. The first type is to repair the missing pixels by relevant data of other time scales under the same spatial conditions^60^. In the second category, under the same time conditions, the missing pixels can be repaired by relevant data from other spatial scales^61^. The third type uses other types of data in the same area at the same time to repair the missing pixels, such as ground site data, microwave data, and other reanalysis data^62^. In this study, the monthly LST data in North America from 2002 to 2018 were obtained by preprocessing MODIS LST products, including data set extraction, coefficient conversion, cropping, projection conversion and other processes. On this basis, we used the quality control data set in the original MODIS LST data to evaluate the quality. We regarded the accuracy to be less than 2 K and the missing information pixels as the area to be repaired for the monthly data. Then, the pixels whose accuracy was less than 2 K and whose information was missing in the daily LST data controlled by this region were set as invalid pixels. After determining the invalid pixels, we repaired and interpolated them. We first assigned the ground station data to the invalid pixels of the corresponding time scale according to the longitude and latitude coordinates and then used the adjacent value substitution method (Nibble) based on the elevation to interpolate and repair the remaining invalid pixels. Finally, we verified the accuracy of the reconstructed data by using the monthly LST data from ground stations. The complete data repair process is detailed in the Fig. 7.

The method of substitution of an adjacent pixel value adopted in this study used the pixel value of an adjacent region on the same time scale for substitution repair. The algorithm first identified an invalid pixel in the image, then calculated its Euclidean distance to the adjacent valid pixel, and finally assigned the nearest valid pixel value to the corresponding invalid pixel. This method is based on the regional consistency principle of geography, which holds that the closer that regions are, the more similar their geographical environments will be. Before using this method for interpolation, we used the ground station data to interpolate some pixels, so the rest of the invalid pixels were sporadically distributed. In this case, we can guarantee the interpolation accuracy in the process using the method of adjacent value substitution. To further improve the accuracy of restoration, we considered the influence of altitude on LST. There have been many studies on the relationship between temperature and altitude in geography. For example, Barry studied the relationship between mountain temperature and altitude^63^. Körner redetermined the geographical significance of altitude and discussed the influence of altitude on temperature^64^. The interpolation method used in this study is a generally accepted rule of temperature vertical decline of 0.55 °C for every 100 m of elevation increase with other conditions unchanged^63^. According to this rule, we first restored the original LST pixel value to the pixel value when the altitude is 0 m. On this basis, the invalid pixels were interpolated by the method of adjacent pixel value substitution. Finally, according to this rule, the interpolated pixel value was restored to the LST of the corresponding elevation. The calculation process was shown in Eqs. (2 and 3). The vertical decline rule of the temperature decreasing by 0.55 °C when the altitude increases by 100 m is applicable to most areas. Therefore, this method can improve the accuracy of LST data reconstruction to a certain extent for pixels with large elevation differences when the distance is short.

2 $$LS{T}_{0m}=LST+0.55\ast (ele/100)$$

3 $$LS{T}_{{\rm{ele}}}={{\rm{LST}}}_{{\rm{nibble}}}-0.55\ast ({\rm{ele}}/100)$$

In the equations, ele is the elevation of the pixel and its unit is m; LST is the MODIS daily LST data; LST_0m_ is the LST at an altitude of 0 m; LST_nibble_ is the interpolation result after substitution of adjacent pixels; and LST_ele_ is the LST restored to the corresponding elevation.

### Spatial and temporal variation analysis methods

To reveal the interannual variation trend of LST from 2002 to 2018, we use the least square method to calculate the interannual variation rate, which represents the average annual variation range of LST from 2002 to 2018. At the same time, we defined the rate of change of the two-tailed t test passing the 0.05 confidence interval as a significant linear change, and we focused on the regions of these significant changes.

To reveal the driving factors of the LST space, we first analyzed the correlation between LST and the NDVI, SM, AOD, cloud fraction and atmospheric WV. The Pearson coefficient is used again to show this correlation. The areas with the absolute value of Pearson coefficient greater than 0.6 were defined as strongly correlated. Correlation analysis can only show the correlations between LST and other parameters but cannot reveal the influence of various parameters on LST. Therefore, on the basis of correlation analysis, we studied the regression relationships between LST and other parameters. By means of a linear multiple stepwise regression model, we reveal the influence of latitude, altitude, the NDVI, SM, AOD, cloud fraction and atmospheric WV on LST. Multiple stepwise regression is an important method in regression analysis. This method is mainly used to determine the number of independent variables from a large number of factors to be selected and to select independent variables to establish the equation with the best regression relationship. Finally, we used RMSE (Root Mean Square Error) and NRMSE (Normalized RMSE) to analyze the error accuracy of the regression model. The methods used in our study for trend analysis, correlation analysis, and regression analysis were all based on the a statistical book written by Wilks, which is named Statistical Methods in the Atmospheric Sciences,100^65^.

### Accuracy verification

Before the spatiotemporal analysis, we verified the accuracy of the reconstructed data. We randomly selected ground stations and verified the accuracy of the reconstructed data by using monthly LST data from ground stations. The verification results show that the average accuracy error between the reconstructed monthly LST data and the ground stations is approximately 1 K-2 K, and the R^2^ value is above 0.95. The accuracy of the data meets the requirements of large-scale spatiotemporal analysis. The specific accuracy verification results were shown in the Supplementary Information.

## Supplementary information

Supplementary information.

## Data Availability

The reconstructed remote sensing LST data set that support the findings of this study are available at 10.5281/zenodo.3529456. The station data we used for data recovery are from the National Oceanic and Atmospheric Administration’s National Center for Environmental Information (NCEI) website. Remote sensing data about the land surface and atmosphere used for the analysis are available on the website of NASA, ESA and JAXA. The large index of climate activity used in the study is available on the America National Climate Center website.
