# Supplementary material for: Driving forces of land surface temperature anomalous changes in North America in 2002–2018
Source: Sci Rep. 2020 Apr 24;10:6931. doi: 10.1038/s41598-020-63701-5 (PMC7181863; doi:10.1038/s41598-020-63701-5)
Supplement: Supplementary file 1 — Supplementary information. [file 41598_2020_63701_MOESM1_ESM.docx]

# Driving forces of land surface temperature anomalous changes in North America in 2002-2018

Yibo Yan^1,^†, Kebiao Mao^1,2,^ †, Jiancheng Shi^3^, Shilong Piao^4^, Xinyi Shen^5^, Jeff Dozier^6^, Yungang Liu^2^, Hong-li Ren^7^, Qing Bao^8^

1. Institute of Agricultural Resources and Regional Planning, Chinese Academy of Agricultural Sciences, Beijing 100081, China; Email: 82101186220@caas.cn

2. School of Geography, South China Normal University, Guangzhou 510631, China; ygliu@scnu.edu.cn

3. State Key Laboratory of Remote Sensing Science, Institute of remote sensing and Digital Earth Research, Chinese Academy of Science and Beijing Normal University, Beijing 100086, China; Email: shijc@radi.ac.cn
4. College of Urban and Environment Sciences, Peking University, Beijing, 100871, China; [slpiao@pku.edu.cn](mailto:slpiao@pku.edu.cn)

5.Civil and Environmental Engineering, University of Connecticut, Storrs, CT 06269, USA; [xinyi.shen@uconn.edu](mailto:xinyi.shen@uconn.edu)

6. Bren School of Environmental Science & Management, University of California, Santa Barbara CA 93106-5131,USA; dozier@ucsb.edu

7. Laboratory for Climate Studies & CMA-NJU Joint Laboratory for Climate Prediction Studies, National Climate Center, China Meteorological Administration, Beijing 100081, China. renhl@cma.gov.cn

8.Institute of Atmospheric Physics, Chinese Academy of Sciences, Beijing, 100029, China. [baoqing@mail.iap.ac.cn](mailto:baoqing@mail.iap.ac.cn)

* Correspondence: maokebiao@caas.cn; Tel.: +86-10-8210-8769.

† These authors contributed equally to this work and should be considered co-first authors.

# Supplementary Method

## Detailed data repair process

We repaired the pixels of missing information and low precision in MODIS monthly LST data. First, the quality control data set^1,2^ was used to select the regions to be repaired in MODIS monthly products. Then, the pixels of missing information and poor precision in MODIS daily LST controlled by this regions are all set as pixels to be repaired. On this basis, the daily data are firstly interpolated by the ground station LST, and then the method of Nibble based DEM was used to repair other pixels. Finally, we averaged daily LST data to generate monthly LST data. The distribution of ground stations is shown in Fig 1.

Accuracy verification

MODIS LST has been verified by many experts and scholars^3,4, 5,6^. We used ground station data to verify the accuracy of reconstructed data in different months. We found that the accuracy errors of LST data in different months were different. The accuracy and integrity of the data have been improved to better meet the accuracy requirements of spatio-temporal analysis. The accuracy verification results of different months are shown in Fig 2.

The spatial difference of LST

## The spatial difference of LST in different seasons in North America was presented Fig 3. We calculated some indicators of seasonal LST. (Table 2)

## Analysis of driving factors

In studying the drivers of LST in North America, we consider two main factors. On the one hand, land surface and atmospheric conditions affect the spatio-temporal variation of LST. On the other hand, large-scale climate activities have a great influence on LST. In the analysis of land surface and atmospheric conditions, we mainly analyzed the impact of five factors including NDVI, Soil Moisture, AOD, Cloud Fraction and Atmospheric WV^7,8,9^. The average status of these five factors in 2002-2018 are shown in Fig 4. In terms of ocean activities, we mainly selected three phenomena closer to North America, including El Nino & La Nina, NAO and PDO^10,11,12^. The climate activity index we used are detailed in Table 1. In our study, various factors were used to simulate and analyze the LST, and the regression model and accuracy were shown in Table 3. The sample points, verification points and different study areas are shown in Fig 5.

# Supplementary Figures


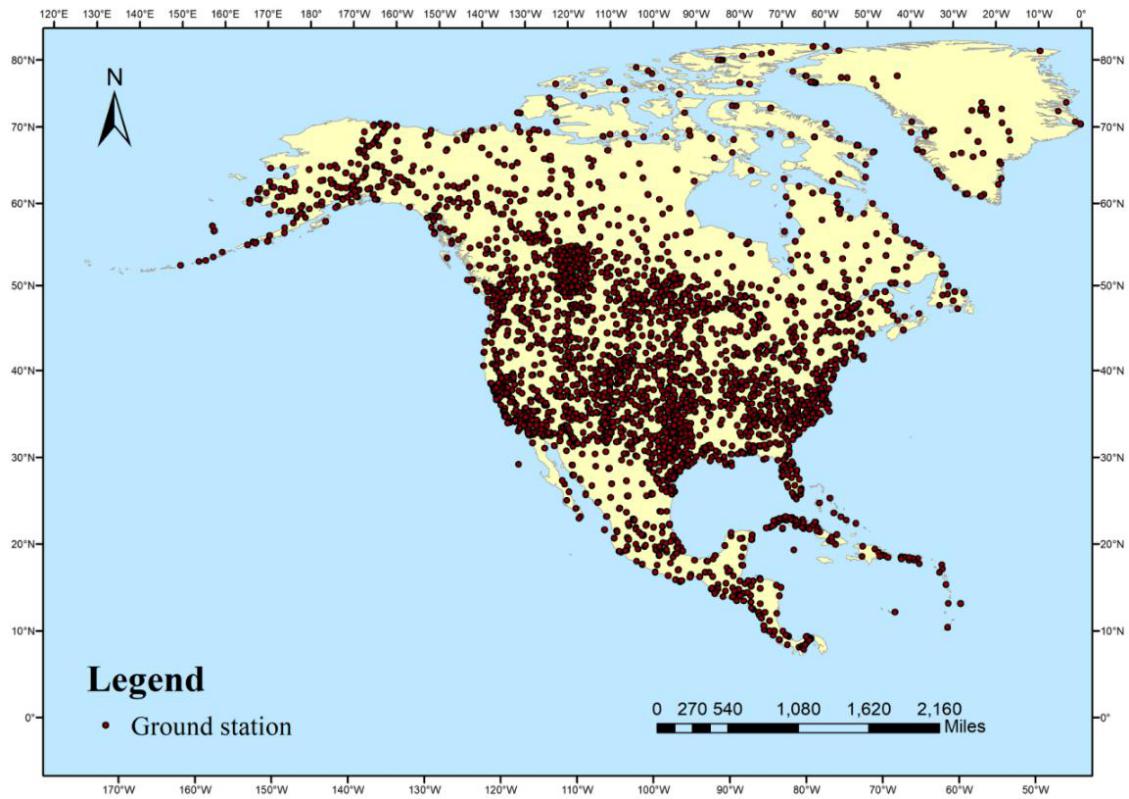


Fig 1. Distribution of ground stations


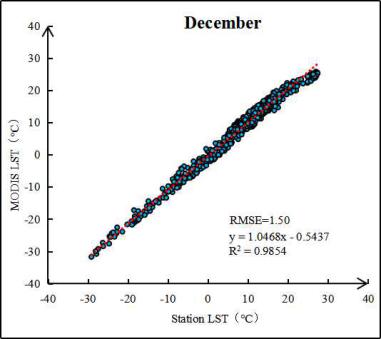

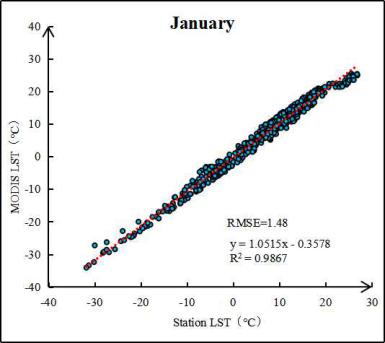

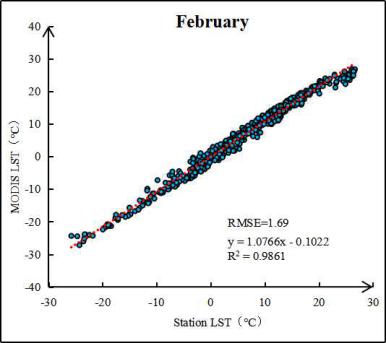


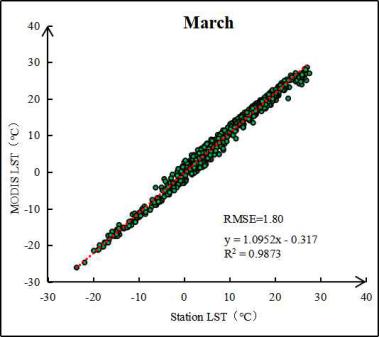

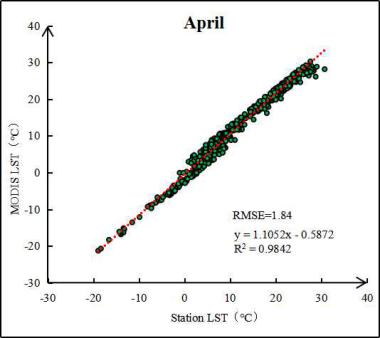

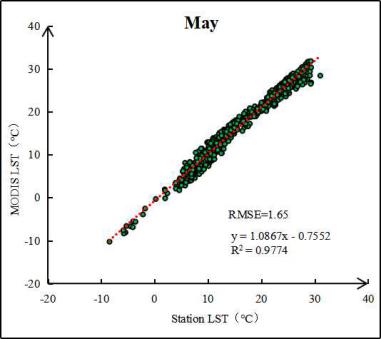


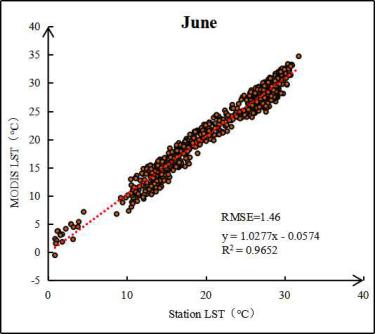

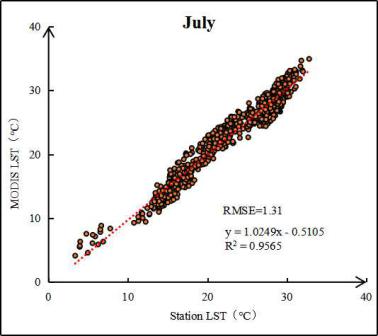

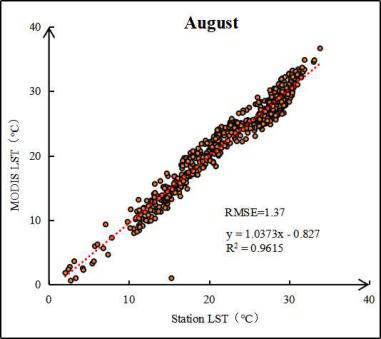


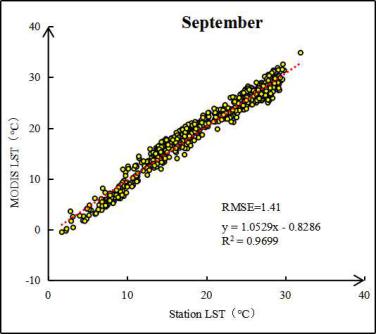

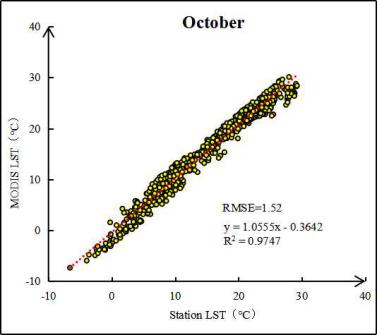

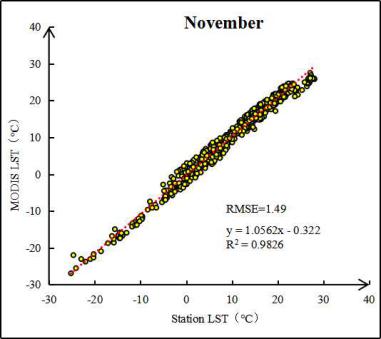


Fig 2. Accuracy verification of reconstructed data in different months. (The same color indicates the precision evaluation in the same season.)


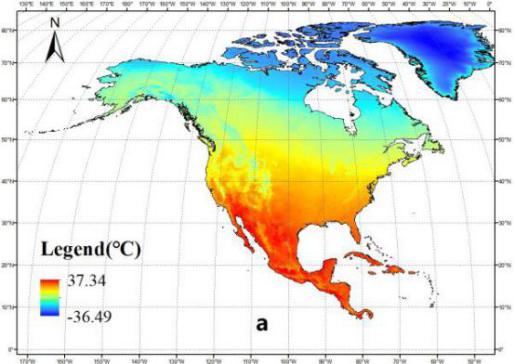

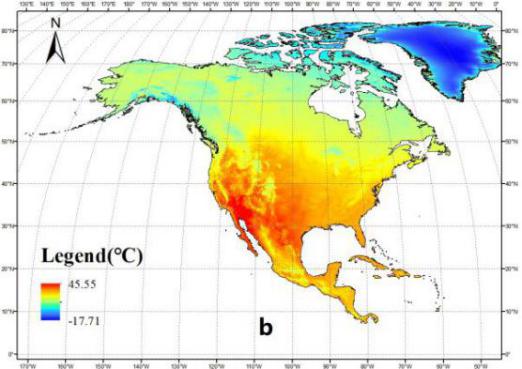


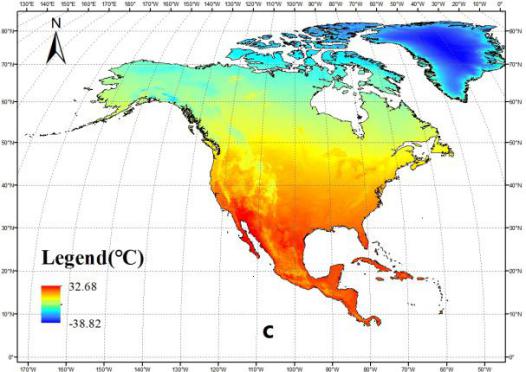

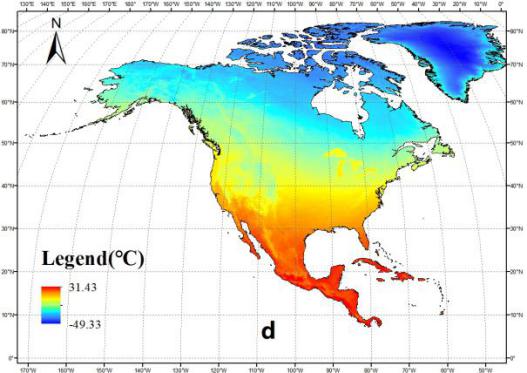


Fig 3.Average LST of North America in different seasons from 2002 to 2018

(**a**,spring. **b**,summer. **c**,autumn. **d**,winter.)


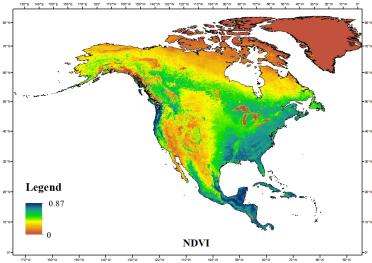

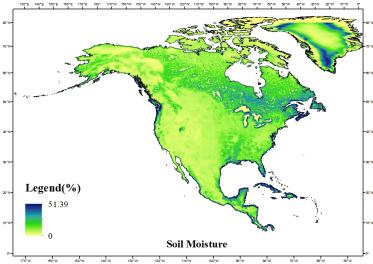

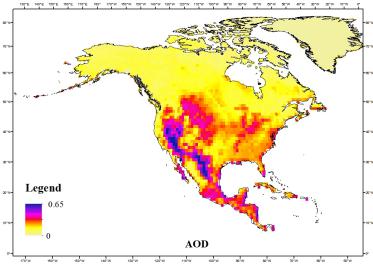


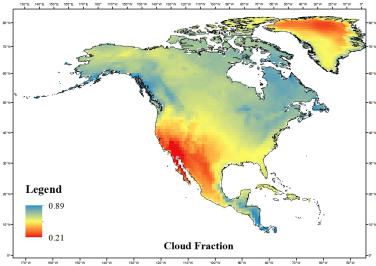

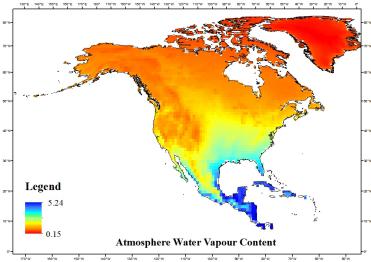


Fig 4 .Average of parameters about land surface and atmospheric from 2002 to 2018.


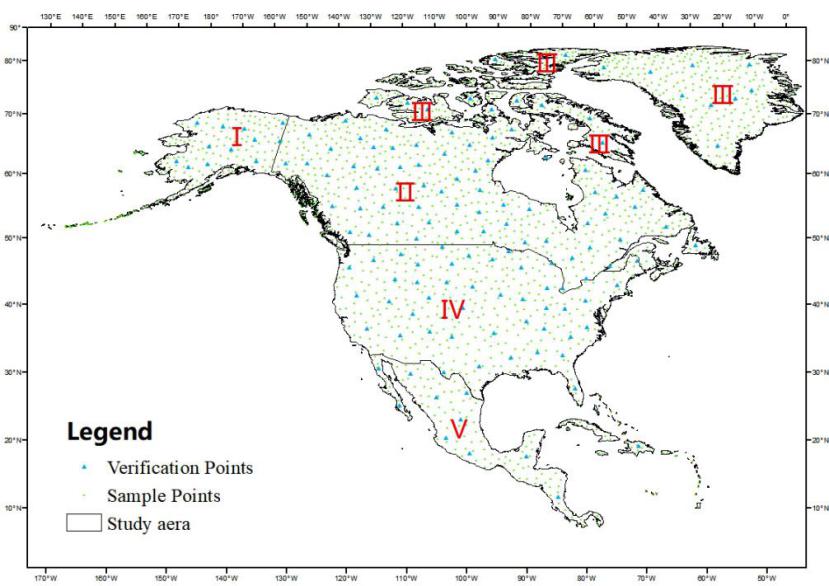


Fig 5. Sample points for LST simulation and sample points for verification of simulation LST

# Supplementary Tables

Table 1 . Introduction of ocean activities index

| Name | Source | Time Scale | Time Frame |
| --- | --- | --- | --- |
| NINO3 Index | NCPC | Month | 2002-2018 |
| North Atlantic Oscillation Index | NCPC | Month | 2002-2018 |
| Pacific Decadal Oscillation Index | NCPC | Month | 2002-2018 |

Table 2 .Statistical indicators of mean LST in different seasons

|  | Spring | Summer | Autumn | Winter |
| --- | --- | --- | --- | --- |
| Average(℃） | 1.03 | 15.72 | 2.63 | -11.62 |
| Maximum(℃） | 37.34 | 45.55 | 32.67 | 31.43 |
| Minimum(℃） | -36.49 | -17.61 | -38.82 | -49.33 |
| Range(℃） | 73.83 | 63.16 | 70.99 | 80.76 |
| Standard deviation | 16.47 | 11.15 | 15.01 | 17.75 |

Table 3. Details of regression mode

| Types  Area | Latitude | DEM | NDVI | SM | AOD | Cloud | Atmospheric WV | R^2^ |
| --- | --- | --- | --- | --- | --- | --- | --- | --- |
| Ⅰ | -0.606 | \ | 0.375 | 0.075 | \ | \ | 0.162 | 0.912 |
| Ⅱ | -0.273 | 0.108 | 0.340 | -0.112 | 0.138 | \ | 0.411 | 0.917 |
| Ⅲ | -0.347 | -0.78 | -0.195 | \ | \ | \ | 0.235 | 0.857 |
| Ⅳ | -0.505 | -0.385 | -0.103 | \ | 0.091 | -0.450 | 0.101 | 0.921 |
| Ⅴ | -0.286 | -0.805 | -0.507 | \ | \ | -0.542 | 0.394 | 0.802 |
| Whole | -0.755 | -0.183 | 0.148 | -0.062 | 0.167 | -0.087 | 0.183 | 0.948 |

# Supplementary References

1. Ke L , Ding X , Song C . Reconstruction of Time-Series MODIS LST in Central Qinghai-Tibet Plateau Using Geostatistical Approach[J]. IEEE Geoscience and Remote Sensing Letters, 2013, 10(6):1602-1606.
2. Markus M , Andreo Verónica, Markus N . A New Fully Gap-Free Time Series of Land Surface Temperature from MODIS LST Data[J]. Remote Sensing, 2017, 9(12):1333-.
3. Wan Z . New refinements and validation of the MODIS Land-Surface Temperature/Emissivity products[J]. Remote Sensing of Environment, 2008, 112(1):59-74.
4. Wan Z , Zhang Y , Zhang Q , et al. Validation of the land-surface temperature products retrieved from Terra Moderate Resolution Imaging Spectroradiometer data[J]. Remote Sensing of Environment, 2002, 83(1-2):163-180.
5. Wan Z , Zhang Y , Zhang Q , et al. Quality assessment and validation of the MODIS global land surface temperature[J]. International Journal of Remote Sensing, 2004, 25(1):261-274.
6. Wan Z . New refinements and validation of the MODIS Land-Surface Temperature/Emissivity products[J]. Remote Sensing of Environment, 2008, 112(1):59-74.
7. Zhou W , Huang G , Cadenasso M L . Does spatial configuration matter? Understanding the effects of land cover pattern on land surface temperature in urban landscapes[J]. Landscape and Urban Planning, 2011, 102(1):0-63.
8. Amiri R , Weng Q , Alimohammadi A , et al. Spatial–temporal dynamics of land surface temperature in relation to fractional vegetation cover and land use/cover in the Tabriz urban area, Iran[J]. Remote Sensing of Environment, 2009, 113(12):2606-2617.
9. Raynolds M K , Comiso J C , Walker D A , et al. Relationship between satellite-derived land surface temperatures, arctic vegetation types, and NDVI[J]. Remote Sensing of Environment, 2008, 112(4):1884-1894.
10. Ashok K , Behera S K , Rao S A , et al. El Niño Modoki and its possible teleconnection[J]. Journal of Geophysical Research: Oceans, 2007, 112(C11).
11. Hurrell J W , Loon H V . DECADAL VARIATIONS IN CLIMATE ASSOCIATED WITH THE NORTH ATLANTIC OSCILLATION[J]. Climatic Change, 1997, 36(3-4):301-326.
12. Giese, B. S. , & Carton, J. A. Interannual and decadal variability in the tropical and midlatitude pacific ocean. Journal of Climate 12, 3402-3418 (1999).
